# Engineering Surface Chemistry to Enhance Ferroelectric Phase Formation in Ultrathin PVDF-TrFE Films

**DOI:** 10.1021/acs.macromol.5c03532

**Published:** 2026-01-14

**Authors:** Andres Mosquera-Vallin, Arnaud Hemmerle, Jon Maiz, Alberto Alvarez-Fernandez

**Affiliations:** † 202635 Centro de Fisica de Materiales (CFM-MPC), CSIC-EHU, 20018 Donostia - San Sebastian, Spain; ‡ 55536 Synchrotron SOLEIL, L’Orme des Merisiers, Départementale 128, 91190 Saint-Aubin, France; § IKERBASQUE-Basque Foundation for Science, Plaza Euskadi 5, 48009 Bilbao, Spain

## Abstract

The development of flexible, lightweight electronic devices has driven growing interest in ferroelectric polymers, with a focus on poly­(vinylidene fluoride-trifluoroethylene) (PVDF-TrFE) copolymers. These materials offer solution processability, mechanical flexibility, and high remanent polarization, making them well-suited for applications in sensors, nonvolatile memories, and energy harvesters. However, as film thickness is reduced below 50 nm, crystallization becomes increasingly sensitive to interfacial interactions, leading to variations in phase composition and surface morphology. This work investigates how controlled modifications to substrate surface chemistry influence the crystallization behavior of ultrathin PVDF-TrFE films. To this end, polymer brushes of varying polarity were grafted onto silicon oxide substrates to create a systematic gradient in surface energy, spanning from hydrophilic to hydrophobic regimes. PVDF-TrFE copolymers with VDF:TrFE ratios of 80:20, 75:25, and 70:30 were spin-coated onto these surfaces to produce uniform films with thicknesses below 50 nm. Ellipsometry and contact-angle measurements were used to confirm brush coverage and film thickness. Crystalline phase composition was quantified using attenuated total reflectance Fourier-transform infrared spectroscopy (ATR-FTIR) and grazing-incidence wide-angle X-ray scattering (GIWAXS), while atomic force microscopy (AFM) was employed to characterize nanoscale surface topography. Results demonstrate that both brush chemistry and copolymer composition significantly affect β-phase content and crystalline texture. Hydrophobic surfaces consistently promoted superior film coverage, larger crystalline domains, and higher electroactive β-phase content compared to hydrophilic counterparts. These findings provide a detailed framework for controlling phase behavior and morphology in nanoconfined PVDF-TrFE films. By controlling the interface rather than the material itself, this study offers a simple and effective strategy for improving ultrathin ferroelectric films, providing useful design guidelines for flexible electronics and opening new directions for research in nanoscale polymer engineering.

## Graphical abstract

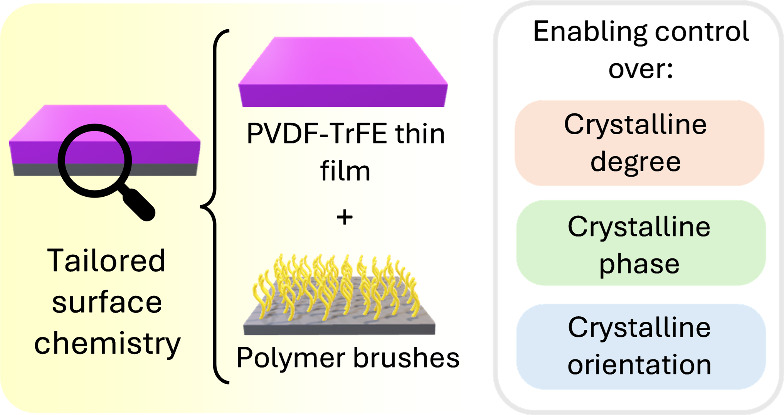

## 1 Introduction

Ferroelectric polymers represent a distinctive class of functional materials that combine switchable electric polarization with the versatility of organic matter. Their spontaneous and reversible dipole alignment enables nonvolatile operation, while their lightweight nature, flexibility, and ease of processing open opportunities unavailable to inorganic ferroelectrics.^1–3^ These features have established them as promising candidates for a broad range of technologies, including nonvolatile memory devices,^4^ piezoelectric and pyroelectric sensors,^5,6^ pressure and vibration detectors,^7–9^ actuators,^10,11^ energy harvesters,^8^ biomedical implants,^12^ and flexible or wearable electronics.^13^ In this context, ultrathin ferroelectric films are particularly critical for such emerging applications, where device miniaturization and mechanical compliance are essential.

Among the various ferroelectric polymers, poly­(vinylidene fluoride) (PVDF) has emerged as particularly important due to its strong ferroelectric response, chemical stability, and compatibility with solution processing.However, PVDF can crystallize into several polymorphs, including the nonpolar α-phase (TGTG′ conformation), the polar β-phase (all-trans conformation), and the less common γ-phase (TTTG′ conformation).^14^ Of these, the β-phase is the most technologically relevant, as its all-trans chain conformation produces the strong net dipole moment required for ferroelectric and piezoelectric performance.^15^ Achieving and stabilizing this phase, however, is nontrivial, since PVDF preferentially crystallizes in the thermodynamically favored α-form under ambient processing conditions. Strategies such as mechanical stretching, application of high electric fields, and the use of nucleating agents have been employed to induce the β-phase.^16–18^ Still, these approaches often add complexity or are difficult to scale. A more effective route is chemical modification, where copolymerization of VDF with trifluoroethylene (TrFE) lowers the energetic barrier for β-phase formation and directly stabilizes the ferroelectric all-trans conformation.^19–21^

The incorporation of TrFE units into the PVDF backbone disrupts the regular TGTG′ conformation that favors α-phase crystallization, instead stabilizing the all-trans arrangement characteristic of the β-phase. This effect arises from the larger steric volume and enhanced electronegativity of the trifluoromethyl group, which promotes dipole−ipole interactions along the chain and reduces the energy barrier for all-trans packing.^22^ As a result, PVDF-TrFE copolymers spontaneously crystallize into the polar β-phase without the need for mechanical stretching or electrical poling, and display robust ferroelectric and piezoelectric behavior even in solution-processed thin films.^23^ Moreover, their ferroelectric properties can be tuned by adjusting the VDF:TrFE ratio, which modifies both the degree of crystallinity and the stability of the ferroelectric phase, making these copolymers highly attractive for flexible electronic and energy-harvesting applications.^24^

PVDF-TrFE copolymers readily stabilize the β-phase in bulk. However, their behavior in the ultrathin-film regime (<50 nm) is far less straightforward.^25^ At these dimensions, confinement effects and interfacial interactions strongly influence crystallization, often reducing overall crystallinity and altering polymorph selection.^26,27^ Consequently, the substrate emerges as a critical determinant of crystallization behavior: its surface energy, chemical functionality, and roughness can either promote or hinder chain alignment and crystal growth.^28,29^ For example, polar oxide surfaces may induce heterogeneous nucleation but also constrain polymer mobility, whereas hydrophobic coatings can facilitate chain spreading during deposition and favor the growth of extended lamellae.^30,31^ Understanding and controlling these substrate–polymer interactions is therefore essential for engineering ultrathin ferroelectric films with well-defined crystalline phases, orientation, and functional properties, thereby enabling high-performance nanoscale devices.

A versatile strategy to control interfacial properties is the use of polymer brushes, where end-grafted polymer chains form a dense, covalently anchored layer on the substrate. Such brushes offer precise control over surface chemistry, wettability, and interfacial energy simply by selecting the appropriate polymer backbone and end group.^32^ Among the various grafting approaches, the thermal condensation of hydroxyl-terminated polymers onto hydroxylated silicon oxide (SiO_2_) surfaces represents a particularly simple, robust, and widely validated method for forming ultrathin polymer brush layers.^33^ In this method, surface silanol (Si–OH) groups generated by UV/ozone or oxygen-plasma activation react with terminal −OH groups of the polymer upon brief thermal annealing (typically 150–200 °C), yielding covalently bound monolayer brushes after removal of nongrafted chains.^34^ This strategy has been successfully demonstrated for a wide range of hydroxyl-terminated polymers, including polystyrene (PS–OH),^34–36^ poly­(2-vinylpyridine) (P2VP–OH),^34,37^ poly­(methyl methacrylate) (PMMA–OH),^38,39^ and related systems, establishing it as a general and reliable route for engineering polymer-functionalized SiO_2_ interfaces. These brushes provide a stable and uniform modification that avoids issues of desorption or phase separation often encountered with physisorbed layers. Despite their broad use in tailoring organic–inorganic interfaces,^40,41^ their application to PVDF-based ferroelectric polymers remains largely unexplored, offering an open and promising route to control nucleation and crystallization at the nanoscale.

In this work, we investigate how interfacial chemistry governs the crystallization and phase behavior of ultrathin PVDF-TrFE films (<50 nm). Silicon substrates were either used in their native oxide form or functionalized with covalently grafted PS brushes to create contrasting hydrophilic and hydrophobic interfaces. Copolymers with different VDF:TrFE ratios were deposited by spin-coating and thermally annealed to promote crystallization. The resulting films were characterized by atomic force microscopy (AFM), attenuated total reflectance Fourier transform infrared spectroscopy­(ATR-FTIR), and grazing-incidence wide-angle X-ray scattering (GIWAXS) to correlate substrate chemistry and copolymer composition with surface morphology, crystalline phase content, and molecular orientation. We hypothesize that substrate surface energy and chemistry, controlled via polymer brush functionalization, can direct the crystallization behavior, phase content, and molecular orientation in ultrathin PVDF-TrFE films. This systematic approach demonstrates that surface functionalization with polymer brushes provides a powerful route to tune ferroelectric polymer ordering and offers new insights into the design of ultrathin ferroelectric films.

## 2 Experimental Section

### 2.1 Materials

Homopolymer polyvinylidene fluoride (PVDF) and copolymer poly­(vinylidene fluoride-trifluoroethylene) (PVDF-*co*-TrFE) with compositions of 70:30, 75:25, and 80:20 were purchased from Piezotech (Arkema), France. Cyclopentanone was purchased from Merck. *N*,*N*-Dimethylformamide (DMF, HPLC grade ≥99.9%) was purchased from Sigma-Aldrich. Tetrahydrofuran (THF, GPC grade) and toluene (>99%) were purchased from Fisher Scientific. Polystyrene hydroxy-terminated (PS–OH, molecular weight (*M*_w_)= 10000 g·mol^–1^, P11116-SOH, PDI: 1.05) was purchased from Polymer Source (Canada). Silicon (100) wafers (p-type) were purchased from MicroChemicals GmbH, cut to appropriate dimensions (1 × 1 cm), and cleaned sequentially with acetone and ethanol under ultrasonic sonication for 10 min each.

### 2.2 Preparation of Hydrophobic Surfaces

Polystyrene with hydroxyl end groups (PS–OH) was used for surface functionalization. The grafting procedure was adapted from the method reported by Lundy et al.^34^ First, a 0.5 wt % PS–OH solution in toluene was spin-coated at 2000 rpm onto cleaned silicon wafers that had been UV/ozone-treated to generate surface −OH groups. The coated substrates were then annealed at 170 °C for 5 min to promote covalent grafting of the PS–OH chain ends onto the hydroxylated surface. After cooling, excess polymer was removed by rinsing with THF.

### 2.3 PVDF-TrFE Ultrathin Film Preparation and Annealing

PVDF-TrFE copolymer and PVDF homopolymer solutions (1 wt %) were prepared by dissolving 0.03 g of polymer in 3.0 g of solvent. Cyclopentanone was chosen as the solvent for the copolymers, whereas PVDF required DMF due to its limited solubility in cyclopentanone. The solutions were then spin-coated (Ossila, UK) onto both hydrophilic and hydrophobic substrates at 1000 rpm for 30 s.

Afterward, a two-step annealing treatment was performed to increase sample crystallinity. The films were first placed on a 160 °C hot plate just long enough to reach that temperature, melting the polymer and erasing any premature nuclei formed during drying. The samples were then rapidly quenched to room temperature to avoid uncontrolled crystallization and immediately transferred to a second hot plate held at 135 °C, where they were maintained for 2 h in an isothermal annealing process. This lower temperature lies within the crystallization window of PVDF-based polymers, promoting controlled nucleation and lamellar thickening, allowing larger, better-oriented crystals grow throughout the film. After annealing, the samples were cooled to room temperature under ambient conditions.

### 2.4 Fourier-Transformed Infrared Spectroscopy (FTIR)

FTIR spectra were recorded on a Jasco FT-IR-6300 spectrometer over the range 1500 to 550 cm^–1^, with a resolution of 4 cm^–1^ and 200 scans per spectrum, using ATR mode at room temperature.

### 2.5 Atomic Force Microscopy (AFM)

All AFM images were acquired with a multimode AFM equipped with a Nanoscope 6 controller (Bruker). The AFM operated in PeakForce Quantitative Nanomechanics (QNM) mode in air at room temperature. Scanning was performed with a silicon tip (PNP-DB, NanoAndMore GmbH, Germany) featuring a spring constant of 0.48 N/m and a resonant frequency of 300 kHz. Images were captured at a scan rate of 1 Hz with a resolution of 512 × 512 pixels, using the Nanoscope software. Subsequent image analysis was carried out using WSxM software.^42^

### 2.6 Thickness Evaluation by Ellipsometry

Ellipsometric measurements were acquired using an EP4 Imaging Ellipsometer (Park Systems) operating at a wavelength of 628 nm, with variable incident angles ranging from 45° to 57°. The data were analyzed and fitted using the Model Studio software from Park Systems.

### 2.7 Grazing-Incidence Wide-Angle X-ray Scattering (GIWAXS)

Experiments were carried out at room temperature on the SIRIUS beamline of the SOLEIL synchrotron (Saint-Aubin, France).^43^ The incident X-ray energy was set to 10 keV (λ = 1.24 Å) using a Si(111) double-crystal monochromator. The beam was defined to a size of 500 × 100 μm^2^ (H × V) and a beam stop was employed to block the direct and reflected beams. Two-dimensional (2D) scattering patterns were recorded on a DECTRIS PILATUS3 1 M detector positioned 310 mm downstream of the sample. The sample-to-detector distance was calibrated using the diffraction pattern of silver behenate. For the data presented, the incidence angle was set at 0.1°, below the total external reflection critical angle of the silicon substrate. Data acquisition involved the integration of 11 frames with 10 s exposures each.

## 3 Results

Figure 1 summarizes the methodology proposed in this work. Ultrathin PVDF-TrFE films (<50 nm) were deposited by spin-coating onto pristine silicon and PS-grafted substrates, followed by thermal annealing to promote crystallization. The resulting films were characterized using ATR-FTIR to assess crystallinity and phase composition, GIWAXS to determine crystalline orientation, and tapping-mode AFM to resolve nanoscale morphology and domain organization.

**Figure 1:**
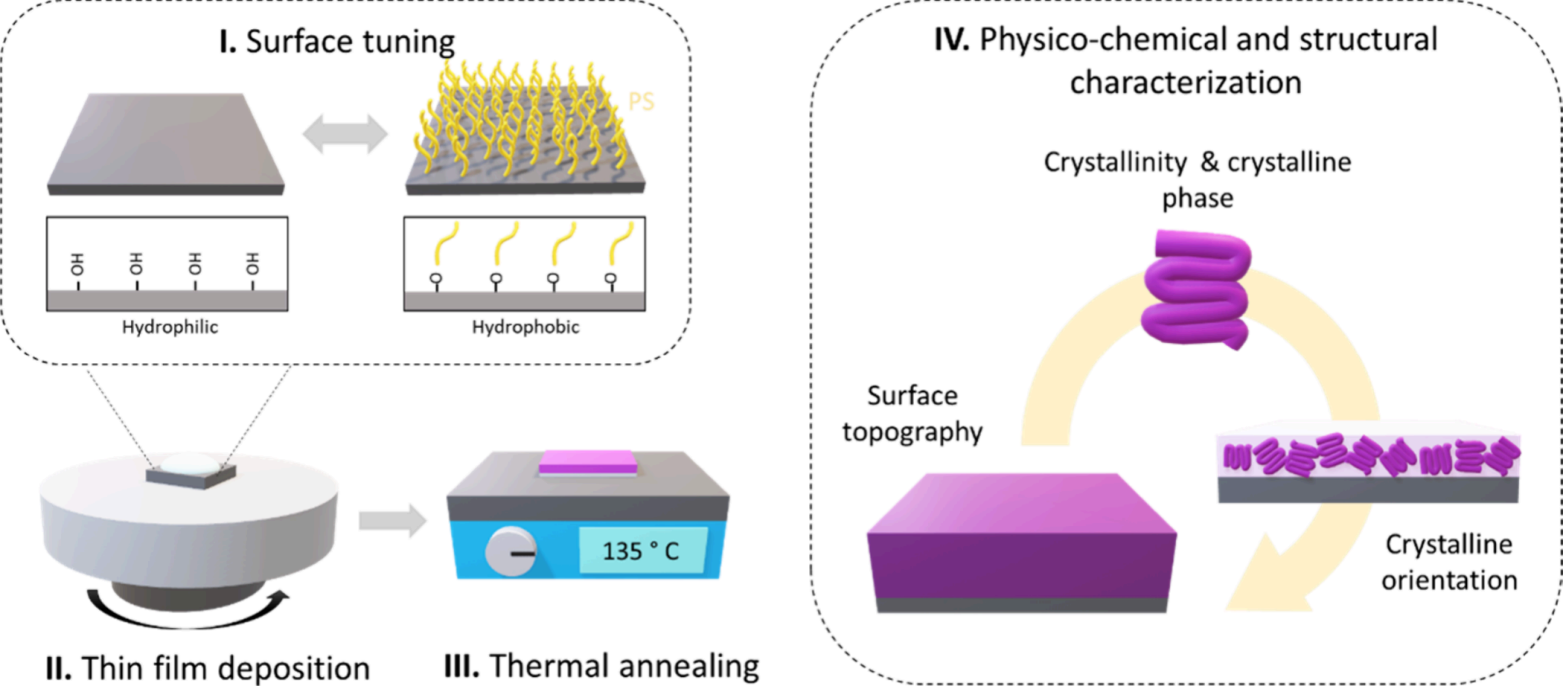
Schematic of the methodology proposed in this work: surface functionalization of silicon substrates, spin-coating of ultrathin PVDF-TrFE films (<50 nm), thermal annealing, and structural characterization.

### 3.1 Engineering Surface Chemistry

Hydrophobic substrates were prepared by grafting PS−OH onto native SiO_2_ surfaces, as described in the Experimental Section. Pristine silicon wafers were spin-coated with a PS–OH solution in toluene and briefly annealed at 170 °C to promote condensation between terminal −OH groups and surface silanols, anchoring the polymer chains to the substrate. Physically absorbed polymer was removed by solvent rinsing and ultrasonication, leaving a covalently bonded PS brush layer (Figure 2A).

**Figure 2:**
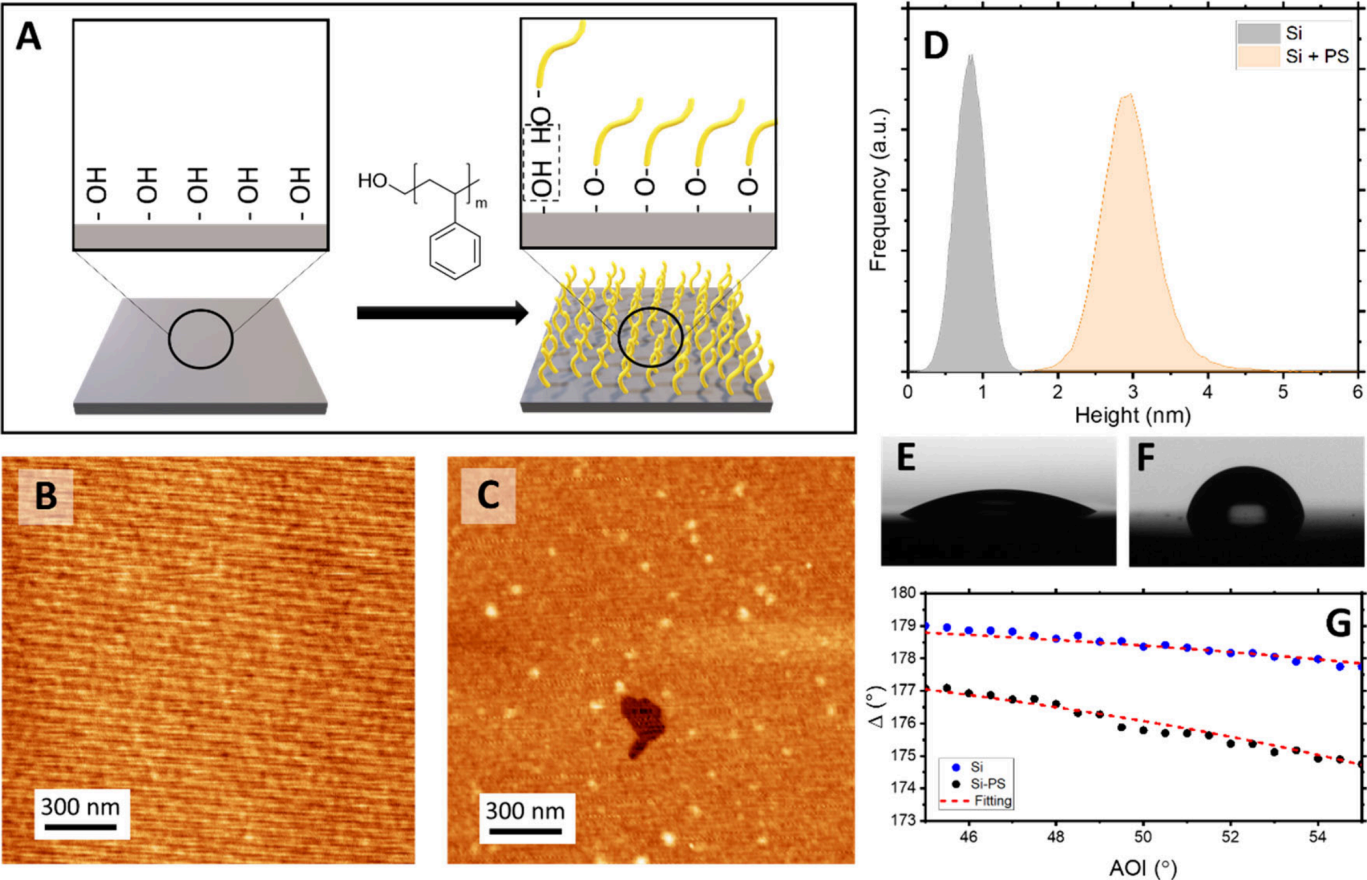
Characterization of PS-grafted silicon substrates. (A) Schematic of the grafting process. AFM images of (B) pristine and (C) PS-grafted silicon. The faint line pattern observed on pristine silicon arises from instrumental scanning noise, due to the subnanometer roughness of polished silicon. (D) Height histograms from AFM, indicating ∼3 nm PS thickness. Water contact-angle images on (E) pristine (∼25°) and (F) PS-modified (∼90°) wafers. (G) Ellipsometry data of the pristine and PS-grafted silicon substrates.

AFM imaging confirmed the formation of a continuous polymer layer with an average thickness of ≈3 nm (Figure 2D), in contrast to the clean, featureless surface of pristine silicon (Figure 2B). The corresponding height histograms further highlight this thickness difference (Figure 2C). Wettability measurements revealed a marked change: the static water contact angle increased from ∼25° for hydrophilic native SiO_2_ (Figure 2F) to ∼90° for PS-grafted wafers (Figure 2G), confirming the reduction in surface energy and the conversion to a hydrophobic surface. Ellipsometry provided additional evidence of successful grafting. Untreated silicon exhibited a native oxide thickness of 2.6 ± 0.3 nm, whereas after PS modification, a clear shift in the Δ curve was observed. Modeling with a Si/SiO_2_/PS stack gave a PS thickness of 2.7 ± 0.3 nm (Figure 2E), in good agreement with AFM observations. The measured brush thickness, wettability, and uniformity are in excellent agreement with values reported in the literature for PS–OH monolayer brushes prepared via thermally activated grafting onto oxidized silicon.^34,36^

### 3.2 PVDF Homopolymer Films

To highlight the importance of substrate surface chemistry, we first investigated the behavior of PVDF homopolymer as a reference system. Ultrathin PVDF films (<50 nm) were deposited onto both pristine silicon and PS-grafted substrates and subsequently annealed. The results reveal a striking difference in wetting behavior and film continuity depending on the underlying surface (Figure S1). On hydrophilic silicon wafers, PVDF displayed poor wetting, leading to discontinuous films with large voids and uncovered areas (Figure S1A). In contrast, deposition on hydrophobic PS-modified wafers yielded a nearly continuous film with improved coverage across the surface (Figure S1B). This difference can be attributed to the intrinsic hydrophobicity of PVDF: on a hydrophobic surface, the polymer wets more effectively and spreads uniformly during spin-coating, whereas poor interfacial compatibility with hydrophilic silicon promotes dewetting and film rupture. Together, these results establish that substrate surface chemistry plays a decisive role in governing PVDF film morphology, providing a useful baseline for understanding the behavior of PVDF-TrFE copolymer films discussed next.

### 3.3 Surface Morphology of PVDF-TrFE Ultrathin Films

Having established a baseline with PVDF homopolymer, we next examined PVDF-TrFE copolymer films to assess how substrate chemistry influences surface morphology. Ultrathin films (<50 nm) with different VDF:TrFE ratios (80:20, 75:25, and 70:30) were deposited on pristine silicon and PS-grafted wafers. Figure S2 presents the AFM topography of spin-cast films prior to annealing. At this stage, the overall wetting behavior appears similar for both substrates, and the films exhibit very low crystallinity, with surfaces dominated by amorphous-like features. Nonetheless, a subtle difference is already apparent: on PS-modified substrates, the residual crystalline domains tend to be slightly larger and better defined than those observed on pristine silicon, suggesting that even before annealing, the hydrophobic interface promotes a marginally more favorable arrangement of polymer chains during deposition.

To verify uniform film formation and determine thickness, ellipsometry was performed on all PVDF-TrFE samples (Figure S3). The raw Δ spectra revealed virtually overlapping curves for films deposited on both pristine and PS-grafted substrates, indicating negligible differences in optical thickness. This behavior can be explained by partial interpenetration of the PVDF-TrFE layer into the grafted PS brush, rather than forming a distinct overlayer. As a result, the system behaves optically as a single, uniform layer, consistent with the miscibility and similar refractive indices of PVDF-TrFE and PS in the interfacial region. Fitting the data with a single-layer optical model yielded thicknesses of 37 ± 2 nm across all samples, confirming that the spin-coating process produces reproducible ultrathin films independent of substrate chemistry or copolymer composition.

Subsequently, to promote crystallization, the films were first melted at 160 °C to erase any residual crystalline fraction and then annealed at 135 °C for 2 h after the quenching process. The resulting AFM images (Figure 3) clearly show a pronounced increase in crystallinity for all compositions and substrates. The dependence on TrFE content is also evident. For the 80:20 composition (Figure 3A,D), the morphology retains a globular crystalline texture, typical of PVDF-rich systems where the high PVDF content favors the formation of compact, isotropic crystalline aggregates rather than well-defined spherulitic structures. As the TrFE fraction increases to 75:25 and 70:30 (Figure 3B,C,E,F), the morphology shifts toward longer, ribbon-like crystalline domains characteristic of PVDF-TrFE copolymers. This transformation reflects the influence of TrFE in increasing chain regularity and stabilizing the all-trans conformation, which favors β-phase formation and the development of more extended crystalline domains with higher aspect ratios.^44^

**Figure 3:**
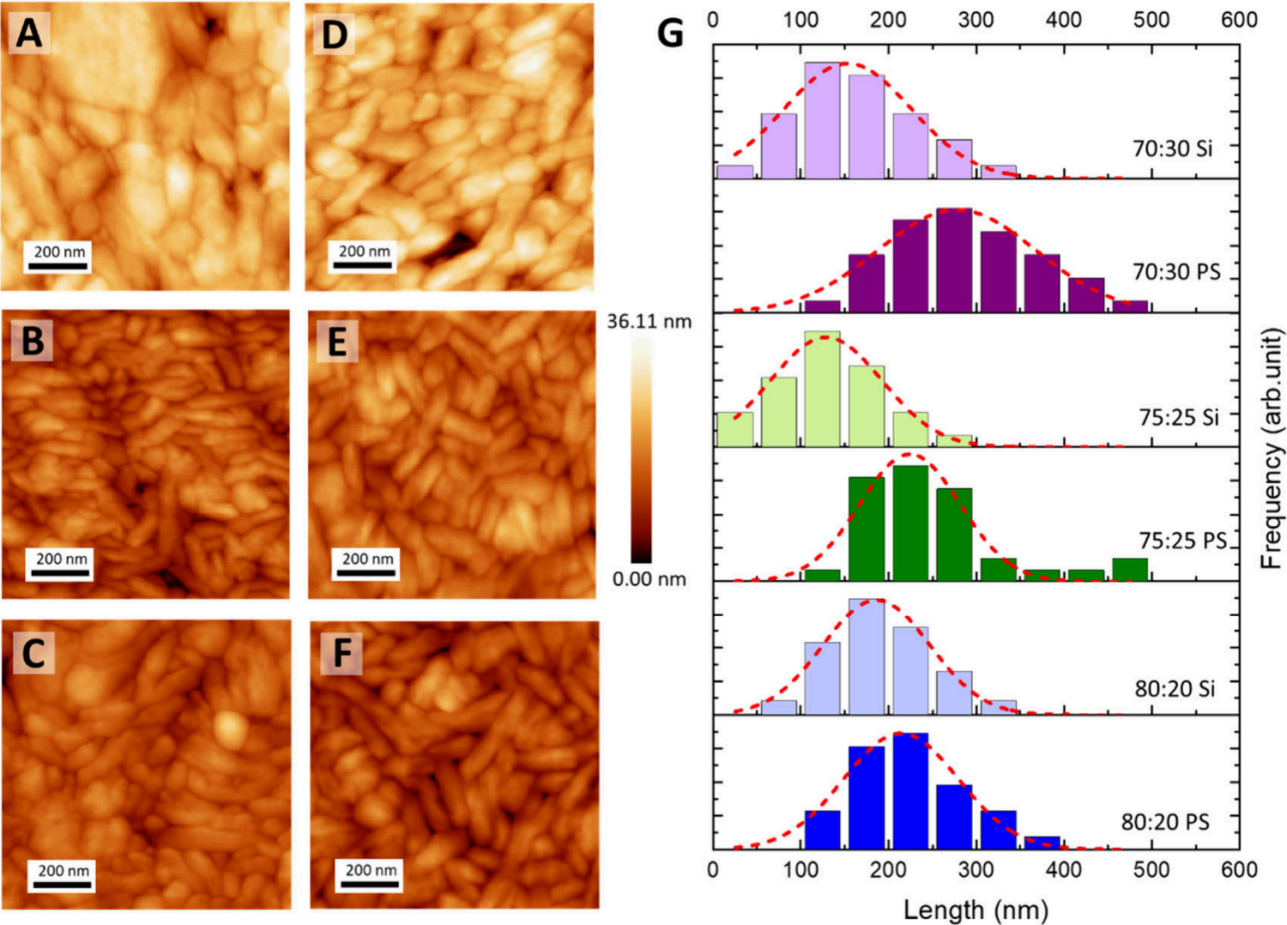
AFM topography images of annealed PVDF-TrFE copolymer thin films with different VDF:TrFE ratios deposited on pristine silicon (A–C) and PS-grafted silicon (D–F): (A, D) 80:20; (B, E) 75:25; (C, F) 70:30. All films were melted at 160 °C to erase residual crystallinity and subsequently annealed for 2 h at 135 °C. (G) Corresponding grain size distributions obtained from the AFM images.

When comparing the two substrates, clear differences emerge. In general, the PS-grafted surfaces promote a more pronounced ribbon-like morphology across all compositions. This effect is particularly evident for the 80:20 copolymer, where the transition from globular to elongated crystalline features is significantly enhanced on PS-modified wafers. For the higher TrFE contents (75:25 and 70:30), the lamellae formed on PS-grafted substrates appear larger and better defined compared to those on pristine silicon, indicating that the hydrophobic interface not only improves film spreading but also favors the development of extended crystalline domains.

These topographical observations are further supported by the adhesion force maps and corresponding histograms (Figures S4 and S5). The contrast observed in the adhesion images closely follows the morphological features seen in the AFM topography, confirming that the brighter, elongated regions correspond to well-defined crystalline lamellae, whereas the darker, more irregular areas are associated with amorphous zones. Importantly, all compositions exhibit systematically lower overall adhesion forces on PS-grafted substrates compared to pristine silicon. In semicrystalline polymer systems, reduced adhesion forces are commonly associated with an increased crystalline fraction, as crystalline domains present lower surface free energy and reduced viscoelastic dissipation compared to amorphous regions. Consequently, the lower adhesion measured on PS-modified substrates indicates a surface dominated by more ordered, crystalline material.^45,46^

To obtain a more quantitative evaluation of the effect of thermal annealing on crystal growth, a systematic size analysis of AFM micrographs was performed. For each copolymer composition and substrate type, the entire AFM image was processed using ImageJ software to extract crystalline domain size distributions, from which average values were calculated as representative metrics (Figure 3G). The resulting histograms reveal clear differences as a function of both TrFE content and substrate chemistry. For PVDF-rich copolymers (80:20), the size distribution is centered around 180 nm on pristine Si and 210 nm on PS-grafted substrates, reflecting the presence of large, globular crystalline domains characteristic of PVDF-rich systems. Increasing the TrFE content to 75:25 and 70:30 results in progressively smaller sizes, indicating a gradual transition to finer and more homogeneous crystalline textures of elongated, ribbon-like lamellae. Specifically, the 75:25 copolymer exhibits mean sizes of 130 nm (silicon) and 224 nm (PS), while the 70:30 composition reaches 150 and 270 nm, respectively. The effect of substrate chemistry is equally pronounced. Across all compositions, films deposited on PS-grafted substrates exhibit larger mean domain sizes and broader distributions than those on pristine silicon, indicating that the hydrophobic interface facilitates the growth and coalescence of extended crystalline lamellae during annealing. This enhancement is most significant for the 75:25 and 70:30 copolymer compositions, where the average size nearly doubles upon moving from silicon to PS, underscoring the strong influence of surface energy in promoting ordered crystallization under conditions already favorable to β-phase formation.

### 3.4 Crystalline Phase of PVDF-TrFE Ultrathin Films

AFM analysis suggested a higher fraction of β-phase in films deposited on PS-grafted substrates, as evidenced by the more pronounced ribbon-like crystalline structures observed after annealing. To verify this morphological indication, ATR-FTIR spectroscopy was performed on all samples, enabling identification of the characteristic vibrational signatures of the α-, β-, and γ-polymorphs and allowing a semiquantitative assessment of β-phase content through peak-area analysis.

Figure 4A–C presents the FTIR spectra of the thermally annealed PVDF-TrFE films, each normalized to the silicon substrate peak at approximately 615 cm^–1^. Vertical bands highlight the main absorption regions corresponding to the different PVDF polymorphs: green for α-, yellow for β-, and orange for γ-. The presence of the β-phase is confirmed by a distinct set of bands at 1400, 1275, 1190, 850, and 750 cm^–1^. The 1400 cm^–1^ feature is attributed to CH_2_ wagging vibration,^47^ while the peaks at 1275 cm^–1^ and 850 cm^–1^ arise from CF_2_ symmetric stretching combined with backbone C–C stretching and C–H bending modes.^48^ The band at 1190 cm^–1^ corresponds to CF_2_ antisymmetric stretching, and the 750 cm^–1^ peak reflects in-plane CH_2_ rocking vibration.^47^

**Figure 4:**
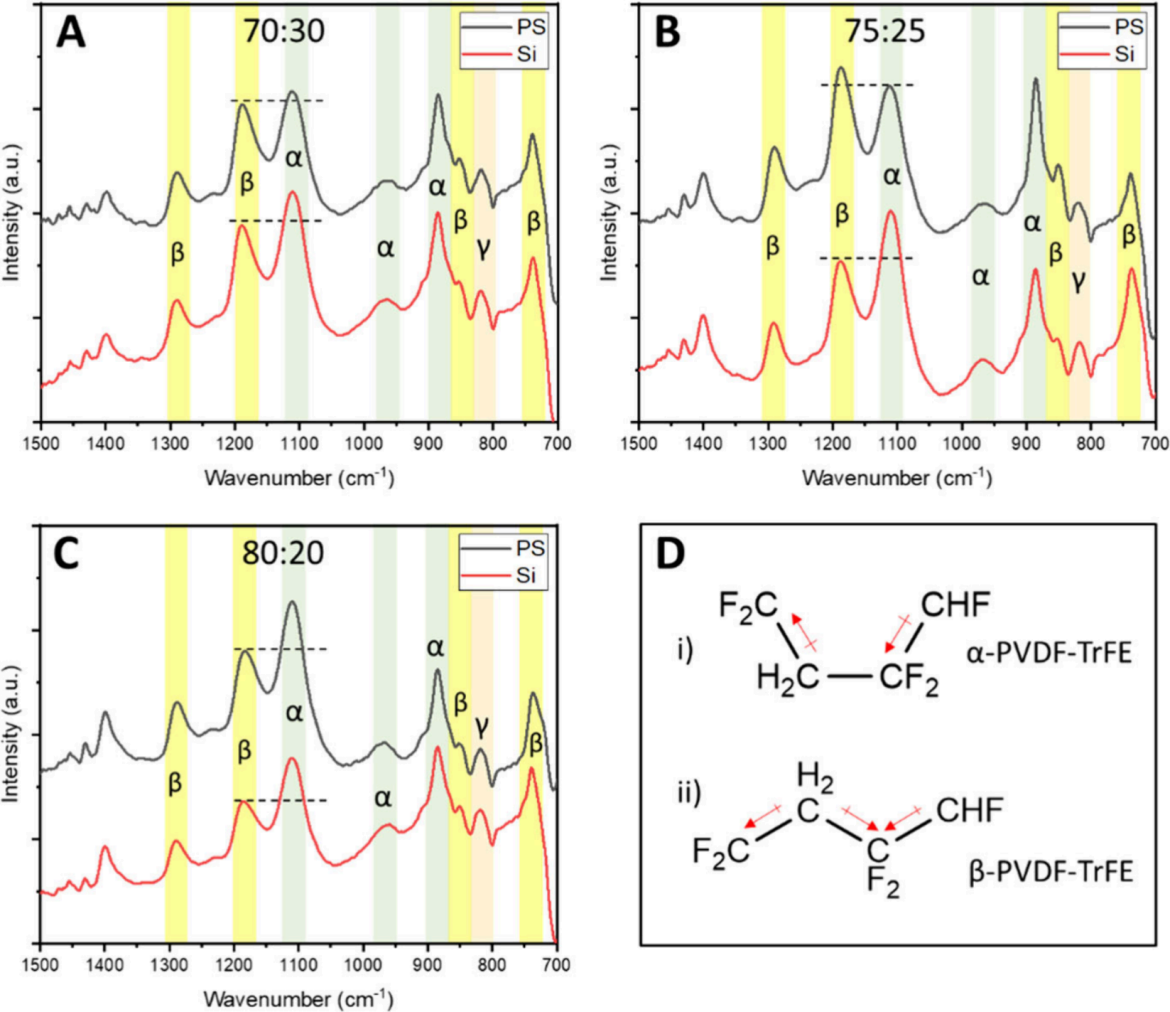
ATR-FTIR spectra of annealed PVDF-TrFE films: (A) 70:30, (B) 75:25, and (C) 80:20 on pristine (red) and PS-grafted (black) silicon surfaces. Highlighted bands indicate α- (green), β- (yellow), and γ- (orange) phase signatures. (D) Schematic representation illustrating chain conformations (all-trans β and TGTG′ α) corresponding to the observed vibrational signatures.

In contrast, the α-phase is identified by absorption bands at 1110, 970, and 880 cm^–1^. The 1110 cm^–1^ peak primarily reflects amorphous contributions but also includes a component associated with C–F stretching specific to the α-phase.^49^ The band at 970 cm^–1^ arises from CH_2_ wagging vibrations in the TGTG′ conformation characteristic of nonpolar α- regions (Figure 4D), while the 880 cm^–1^ feature is attributed to CH_2_ symmetric stretching within that same phase.^48^ A weaker signal near 810 cm^–1^ corresponds to the γ-phase, associated with CF_2_ symmetric stretching in the TTTG conformation. However, the low intensity of this peak indicates that the γ-phase is only a minor component under the present processing conditions.

Across all copolymer compositions and substrates, the presence of vibrational signatures from the α-, β-, and γ-phases confirms that the PVDF-TrFE films exhibit a mixed-phase microstructure. To assess how substrate chemistry influences the α:β balance, three diagnostic vibrational markers were analyzed: the β-specific bands at 1285 and 1190 cm^–1^, and the α-associated band at 1110 cm^–1^.^50^ For each composition (70:30, 75:25, and 80:20), the relative intensities of the 1190 cm^–1^ and 1110 cm^–1^ bands vary significantly with substrate. Films deposited on PS-grafted silicon consistently exhibit stronger β-phase signatures, indicating enhanced β-phase formation compared to those on bare Si. This effect is particularly pronounced for the 75:25 copolymer, where the 1190 cm^–1^ β-band surpasses the 1110 cm^–1^ α-band on PS-modified silicon, whereas the opposite trend is observed on hydrophilic Si/SiO_2_ (Figure 4B). These results suggest that the hydrophobic PS brush promotes β-phase crystallization, likely through improved wetting and spreading during spin-coating. Enhanced chain mobility during annealing at 135 °C enables polymer segments to rearrange into the all-trans β conformation, whereas the hydrophobic–hydrophilic mismatch between PVDF-based copolymers and bare SiO_2_ restricts interfacial chain mobility, hindering reorganization and favoring retention of the nonpolar α-phase.

To quantify the β-phase content, the spectral region from 1050 to 1350 cm^–1^ was analyzed by Gaussian deconvolution, separating the overlapping α-phase band at 1110 cm^–1^ from the β-phase bands at 1190 and 1285 cm^–1^. The 1285 cm^–1^ peak, a robust marker of the all-trans β conformation, was used to compute β-phase fractions.^50^ The fitted curves (Figure 5) provided peak areas enabling direct comparison across samples, with the extracted values summarized in Table 1. Consistently, PS-functionalized substrates promoted higher β-phase formation than pristine silicon. This enhancement is most pronounced for the 70:30 and 75:25 copolymers, where β-band areas increased from 2.49 to 5.86 and from 4.96 to 7.15, respectively, indicating efficient stabilization of the ferroelectric conformation during annealing. The 80:20 copolymer also showed a clear increase on PS surfaces (3.29 vs 4.76), although its absolute β-phase content remained lower.

**Figure 5:**
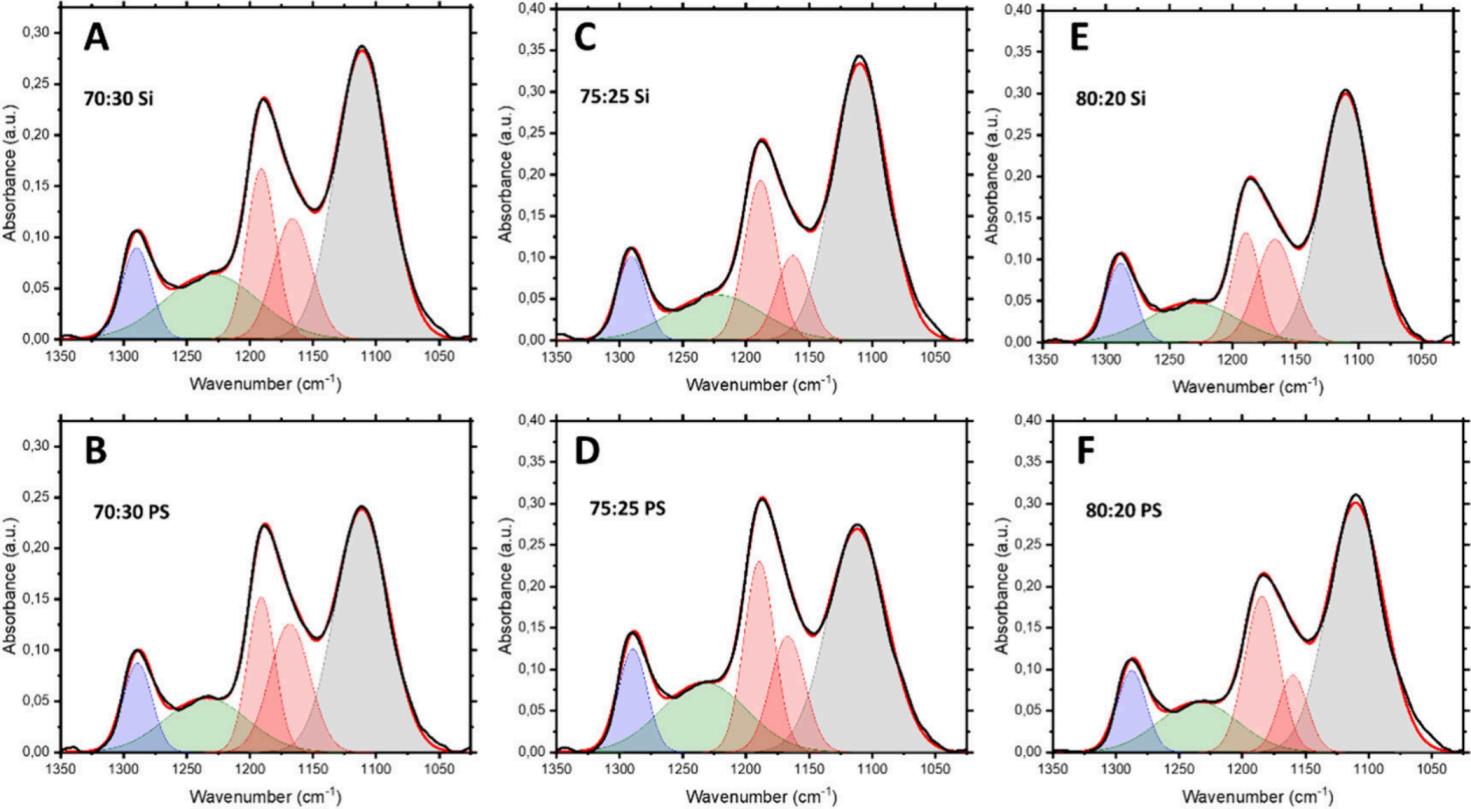
Gaussian deconvolution of the ATR-FTIR spectra in the 1050–1350 cm^–1^ region for PVDF-TrFE copolymer films with different VDF:TrFE ratios: (A, B) 70:30; (C, D) 75:25; and (E, F) 80:20, deposited on pristine (top row) and PS-grafted (bottom row) silicon surfaces.

**Table 1:** Extracted β-Phase Band Areas at 1285 cm^–1^ (from Gaussian Deconvolution of ATR-FTIR Spectra) for PVDF-TrFE Copolymers of Different Compositions Deposited on Si–OH and PS-Modified Si Substrates

	surface type	
composition	Si–OH	Si-PS
70:30	2.49	5.86
75:25	4.96	7.15
80:20	3.29	4.76

### 3.5 Crystalline Orientation of PVDF-TrFE Ultrathin Films

While ATR-FTIR provided qualitative and semiquantitative insight into the phase composition of PVDF-TrFE films, GIWAXS offers a more direct probe of their crystalline structure and molecular arrangement. Figure 6 presents the 2D diffraction patterns of annealed PVDF-TrFE films with different VDF:TrFE ratios deposited on pristine silicon and PS-grafted substrates.

**Figure 6:**
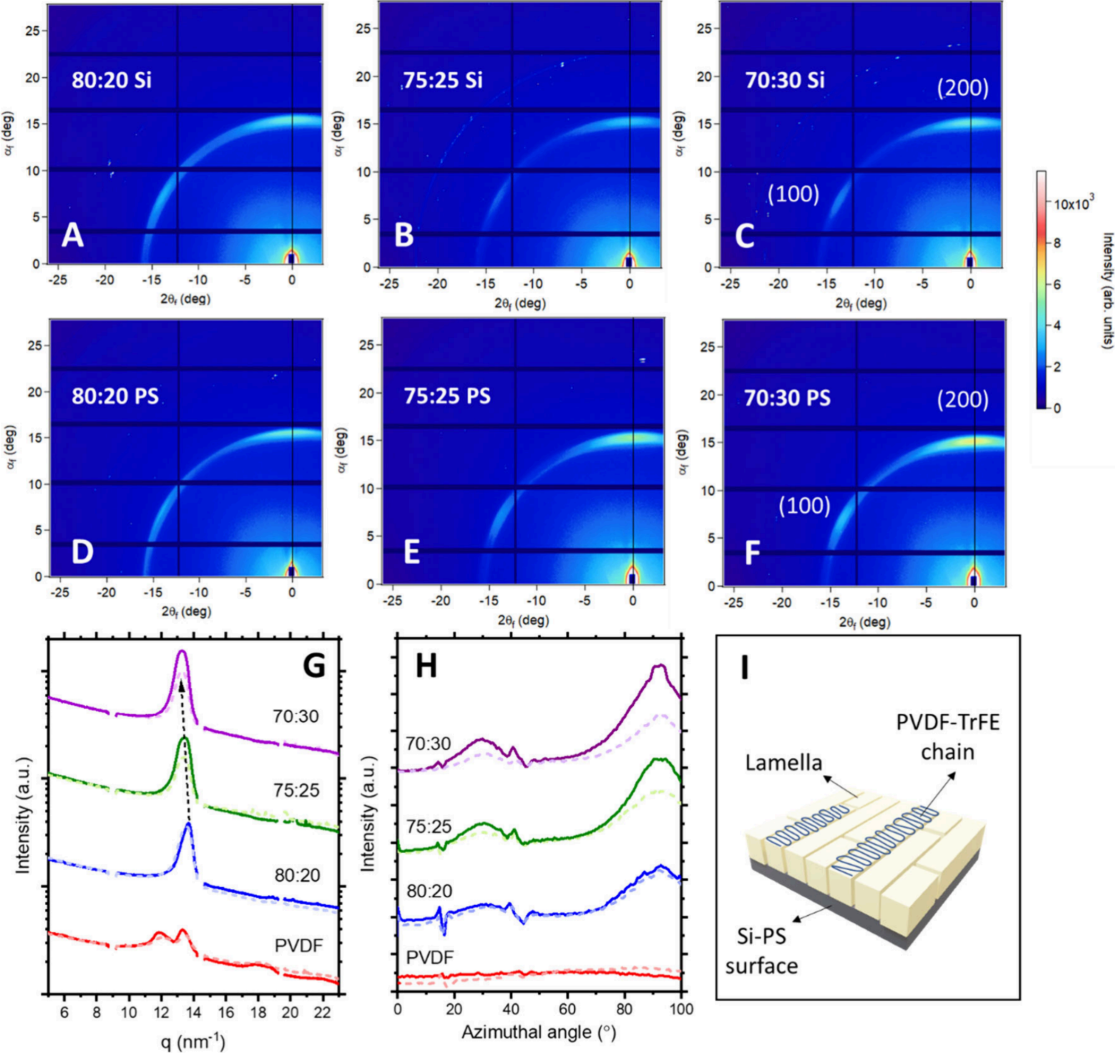
GIWAXS analysis of annealed PVDF-TrFE copolymer films on pristine silicon and PS-grafted substrates. (A–F) 2D GIWAXS patterns for 80:20, 75:25, and 70:30 compositions on silicon (A, C, E) and PS (B, D, F). All films exhibit diffraction peaks characteristic of the β-phase (110/200 reflection at *q* ≈ 14.1 nm^–1^). (G) Radial intensity profiles used to determine crystalline content on silicon (dotted line) and PS-grafted (continuous line). (H) Azimuthal intensity distributions of the β-phase reflection, showing improved orientation in films deposited on PS-modified substrates (continuous line). (I) Schematic showing the edge-on orientations on the PS-modified surfaces.

Radial intensity profiles along *q*_*z*_ (Figure 6G) provide clear evidence of the β-phase dominance in all copolymer films. A single diffraction maximum appears at *q* ≈ 13–14 nm^–1^, corresponding to the overlapping (110)/(200) reflections characteristic of the all-trans β-phase, while neat PVDF shows two distinct peaks at lower *q*, characteristic α-phase peaks assigned as (100)/(110) planes respectively (Figures S6 and 6G).^51^ A systematic shift of the β-phase reflection toward lower *q* with increasing TrFE content indicates a gradual lattice expansion, attributed to the incorporation of bulkier TrFE units within the PVDF crystalline lattice, consistent with previous observations.^44^ Notably, the diffraction intensity is consistently higher for films deposited on PS-grafted substrates than on pristine silicon, pointing to enhanced crystallinity and tighter molecular packing at the hydrophobic interface, an effect consistent with the AFM observations of larger and better-defined lamellae.

Beyond confirming phase composition, the 2D GIWAXS patterns provide valuable insight into the orientational order of the β-phase lamellae. Although all films exhibit the characteristic β-phase scattering ring at *q* ≈ 14 nm^–1^, the intensity distribution along the ring varies markedly with both copolymer composition and substrate. For the PVDF-rich 80:20 copolymer, the intensity is nearly uniform around the azimuth, consistent with a largely isotropic arrangement of crystallites. As the TrFE content increases to 75:25 and 70:30, the scattering becomes progressively anisotropic, with two distinct intensity maxima emerging along the meridional and off-meridional directions. These features correspond to the (200) and (110) reflections, respectively, and indicate the presence of lamellae adopting edge-on orientations, that is, with their lamellar planes parallel to the substrate and polymer chain axes aligned laterally across the surface (Figure 6I).

The anisotropy is significantly more pronounced for films deposited on PS-grafted substrates (Figure 6H). In these samples, the (110)/(200) reflections appear sharper and more localized along the meridian, signifying enhanced lamellar alignment and higher structural coherence. This behavior resembles that reported for PVDF-TrFE films grown epitaxially on oriented PTFE surfaces, where lattice matching promotes edge-on stacking of lamellae along a preferred in-plane direction.^52^ Although no true epitaxy occurs here, the PS brush layer effectively lowers the interfacial energy and reduces the density of random nucleation sites, allowing lamellae to grow laterally in a more ordered and continuous fashion. Conversely, films on pristine silicon show broad, diffuse rings indicative of random or weakly oriented crystallites, confirming that the hydrophobic PS interface acts as a soft template that fosters orientational order.

## 4 Discussion

The results presented above demonstrate that surface chemistry decisively influences the crystallization pathway of PVDF-TrFE in the ultrathin regime. While crystallization in bulk PVDF-based copolymers has been extensively investigated, the mechanisms governing structural evolution in films below ∼50 nm remain far less understood due to the dominant role of substrate–polymer interactions. In this confined geometry, the balance between substrate–polymer, polymer–air, and interfacial enthalpic interactions dictates nucleation density, lamellar orientation, and polymorphic selection.

In this context, the enhanced β-phase formation observed on PS-grafted substrates parallels recent reports of PVDF-TrFE crystallization on low-surface-energy or lattice-matched interfaces, such as fluorinated self-assembled monolayers, PTFE, and graphene, where hard interfacial templating induces pronounced molecular ordering.^53–55^ The epitaxial alignment of PVDF-TrFE lamellae on ordered PTFE layers, for instance, has been shown to arise from partial lattice matching between the (010) plane of PVDF-TrFE and the (100) plane of PTFE, resulting in needle-like lamellae with the *c*-axis parallel to the polymer chain axis of the substrate.^56^ Similar epitaxial correlations have been reported for films on graphene and rubrene single crystals, where small lattice mismatches (<5%) enable preferential orientation of the ferroelectric phase and enhance polarization switching.^57^ These findings highlight how even subtle interfacial registry can direct ferroelectric ordering through local templating effects.^58^

In contrast to these hard, structurally well-defined interfaces the PS brushes used here are amorphous and lack a crystalline lattice. Nevertheless, their low surface energy combined with a soft, deformable interfacial character may mimic certain aspects of epitaxial growth by modulating chain adsorption and promoting lateral lamellar extension. Ellipsometric data (Figure S3) indicating partial interpenetration between PVDF-TrFE and the PS layer support the formation of a diffuse polymer–polymer interface with a gradual mobility gradient, which can facilitate cooperative chain alignment during annealing. This “soft templating” mechanism differs from rigid epitaxial crystallization on hard hydrophobic substrates yet achieves comparable control over lamellar orientation and β-phase enrichment, as reflected in the anisotropic GIWAXS patterns (Figure 6) and ATR-FTIR results (Figure 4). These observations are consistent with previous computational studies of polymer-brush/melt interfaces, which show that grafted brushes act as deformable, soft substrates whose chain mobility and interpenetration with free chains can orient adjacent polymer segments and influence crystallization kinetics.^59^

Beyond lattice and energetic considerations, recent reports have also emphasized that substrate roughness and interfacial friction can also modulate nucleation kinetics and crystal morphology.^60,61^ Thus, on rough or high-energy surfaces (e.g., SiO_2_ or Au), strong adhesion and restricted chain diffusion favor globular crystallites, whereas lower-energy surfaces promote the formation of ribbon-like domains. In our system, these effects are further accentuated by the nanometric confinement and the presence of a compliant PS brush layer. Unlike rigid low-energy substrates, the grafted PS chains introduce a dynamically soft interface that reduces interfacial friction while simultaneously allowing partial chain interpenetration. This dual effect promotes cooperative lamellar reorganization and β-phase stabilization not merely through reduced surface energy, but through a distinct soft-templating mechanism specific to polymer–polymer interfaces.^62^

At the molecular level, these interfacial effects can be rationalized in terms of the conformational energetics of the PVDF-TrFE chains. The stabilization of the β-phase reflects a higher population of all-trans conformers, favored when interfacial constraints reduce torsional disorder and facilitate dipole alignment along the chain backbone.^63^ In confined geometries, the reduced entropic freedom near the substrate and the presence of a soft, low-energy interface can lower the free-energy barrier for trans-conformation nucleation, thereby promoting the ferroelectric phase. This thermodynamic picture is supported by the AFM and GIWAXS results (Figures 3 and 6), which together reveal increased crystal length and enhanced crystalline coherence on PS-modified substrates.

From a broader perspective, these results provide experimental evidence that ferroelectric ordering in ultrathin PVDF-TrFE films can be tuned through interfacial free energy engineering rather than chemical modification of the polymer itself. This finding is particularly relevant for applications where maintaining the intrinsic copolymer composition is critical, such as flexible electronics and nonvolatile memory devices. By decoupling structural control from molecular design, surface functionalization with polymer brushes offers a powerful, scalable approach to manipulate ferroelectric phase stability and orientation.

The microstructural control achieved through interfacial engineering has direct implications for ferroelectric switching behavior. Enhanced β-phase content and lamellar alignment are expected to increase remanent polarization and reduce coercive voltage by enabling more cooperative dipole rotation under an applied field.^64^ This correlation between molecular ordering and macroscopic response has recently attracted renewed attention for flexible ferroelectric devices, where interfacial design offers a scalable route to control polarization without altering copolymer composition.^65^

Overall, these findings reveal that interfacial engineering using polymer brushes provides an alternative paradigm to conventional epitaxial templating for controlling ferroelectric polymer crystallization. The PS-grafted interface combines chemical robustness with mechanical compliance, enabling nanoscale modulation of chain mobility and orientation. This “soft-templating” approach expands the design space for integrating PVDF-TrFE and related semicrystalline polymers into flexible, low-voltage electronic architectures,^65^ where interfacial structure becomes as crucial as molecular composition in defining functional performance.

## 5 Conclusions

This study demonstrates that interfacial chemistry plays a decisive role in directing the crystallization behavior of ultrathin PVDF-TrFE copolymer films. The introduction of covalently anchored polystyrene brushes on silicon surfaces effectively tailored the interfacial energy landscape, enabling the formation of continuous, uniform films even at nanometric thicknesses. Structural characterization by AFM, ATR-FTIR, and GIWAXS collectively revealed that these engineered interfaces promote a pronounced increase in β-phase content, enhanced lamellar connectivity, and improved molecular alignment. Beyond these structural effects, the findings establish that polymer–substrate interactions govern crystallization pathways through a “soft-templating” mechanism unique to polymer–polymer interfaces. The PS brush layer provides a dynamic interfacial region that mediates chain mobility and facilitates cooperative lamellar reorganization, achieving ferroelectric β-phase stabilization without the need for rigid epitaxial constraints. This mechanism extends current understanding of crystallization under confinement, highlighting how nanometric interfacial structure and mobility gradients can dictate polymorphic selection and orientation in semicrystalline polymers. Overall, this interfacial engineering strategy offers a scalable and chemically robust route for controlling ferroelectric ordering in PVDF-based thin films. By coupling molecular composition with interfacial design, it enables targeted optimization of ferroelectric, piezoelectric, and dielectric responses in flexible electronics, sensors, and energy-harvesting devices, where nanoscale structure–property relationships are critical.

## Supplementary Material



## Notes

AFM images of PVDF homopolymer and nonannealed PVDF-TrFE copolymers; Ellipsometric measurements; Adhesion force AFM maps; 2D GIWAXS patterns for PVDF homopolymer (PDF)

## References

[1] Jia X., Guo R., Tay B. K., Yan X. (2022). Flexible Ferroelectric Devices: Status
and Applications. Adv. Funct Mater..

[2] Li L., Han L., Hu H., Zhang R. (2023). A Review on Polymers and Their Composites
for Flexible Electronics. Mater. Adv..

[3] Lee M., Jeong B. (2025). Organic Ferroelectrics
for Regulation of Electronic and Ionic Transport
Toward Neuromorphic Applications. Advanced Physics
Research.

[4] Zhu H., Yamamoto S., Matsui J., Miyashita T., Mitsuishi M. (2018). Resistive
Non-Volatile Memories Fabricated with Poly­(Vinylidene
Fluoride)/Poly­(Thiophene) Blend Nanosheets. RSC Adv..

[5] Zhang M., Tan Z., Zhang Q., Shen Y., Mao X., Wei L., Sun R., Zhou F., Liu C. (2023). Flexible Self-Powered Friction Piezoelectric
Sensor Based on Structured PVDF-Based Composite Nanofiber Membranes. ACS Appl. Mater. Interfaces.

[6] Li L., Peng F., Zheng G., Dai K., Liu C., Shen C. (2023). Electrospun Core-Sheath PVDF Piezoelectric
Fiber for Sensing Application. ACS Appl. Mater.
Interfaces.

[7] Khattak M. M., Headings L. M., Dapino M. J. (2023). Dynamic Response of a Polyvinylidene
Fluoride (PVDF) Sensor Embedded in a Metal Structure Using Ultrasonic
Additive Manufacturing. Actuators.

[8] Maria
Joseph Raj N. P., Ks A., Khandelwal G., Kim S. J. (2022). Method for Fabricating Highly Crystalline Polyvinylidene
Fluoride for Piezoelectric Energy-Harvesting and Vibration Sensor
Applications. Sustain Energy Fuels.

[9] Du H., Zhang N., Xiong B., Zhang X., Yuan X. (2023). High Performance
Flexible PVDF Film Pressure Sensor Fabricated by Femtosecond Laser. Opt Laser Technol..

[10] Pan J., D’Anniballe R., Carloni R. (2025). Rolled Soft Actuators Based on P­(VDF-TrFE-CTFE)
Electrospun Nanofibers. Advanced Intelligent
Systems.

[11] Thuau D., Kallitsis K., Dos Santos F. D., Hadziioannou G. (2017). All Inkjet-Printed
Piezoelectric Electronic Devices: Energy Generators, Sensors and Actuators. J. Mater. Chem. C Mater..

[12] Concha V. O. C., Timóteo L., Duarte L. A. N., Bahú J. O., Munoz F. L., Silva A. P., Lodi L., Severino P., León-Pulido J., Souto E. B. (2024). Properties, Characterization and
Biomedical Applications of Polyvinylidene Fluoride (PVDF): A Review. J. Mater. Sci..

[13] Tian G., Tang L., Zhang J., Wang S., Sun Y., Ao Y., Yang T., Xiong D., Zhang H., Lan B., Deng L., Deng W., Yang W. (2023). Ultrathin Epidermal
P­(VDF-TrFE) Piezoelectric Film for Wearable Electronics. ACS Appl. Electron Mater..

[14] Cui Z., Hassankiadeh N. T., Zhuang Y., Drioli E., Lee Y. M. (2015). Crystalline
Polymorphism in Poly­(Vinylidenefluoride) Membranes. Prog. Polym. Sci..

[15] Martins P., Lopes A. C., Lanceros-Mendez S. (2014). Electroactive
Phases of Poly­(Vinylidene
Fluoride): Determination, Processing and Applications. Prog. Polym. Sci..

[16] Yasar M., Hassett P., Murphy N., Ivankovic A. (2024). β Phase
Optimization of Solvent Cast PVDF as a Function of the Processing
Method and Additive Content. ACS Omega.

[17] Cai X., Lei T., Sun D., Lin L. (2017). A Critical Analysis of the α,
β and γ Phases in Poly­(Vinylidene Fluoride) Using FTIR. RSC Adv..

[18] Fathollahzadeh V., Khodaei M. (2024). Enhanced Piezoelectric
Response of PVDF by Incorporating
of BaTiO3 Nanoparticles and Surface Treatment. Journal of Materials Science: Materials in Electronics.

[19] Kallitsis K., Soulestin T., Tencé-Girault S., Brochon C., Cloutet E., Domingues
Dos Santos F., Hadziioannou G. (2019). Introducing
Functionality to Fluorinated Electroactive Polymers. Macromolecules.

[20] Kallitsis K., Alvarez-Fernandez A., Cloutet E., Brochon C., Hadziioannou G. (2024). Introducing
Photo-Cross-Linkable Functionalities on P­(VDF-Co-TrFE) Ferroelectric
Copolymer. ChemPlusChem..

[21] Spampinato N., Maiz J., Portale G., Maglione M., Hadziioannou G., Pavlopoulou E. (2018). Enhancing
the Ferroelectric Performance of P­(VDF-Co-TrFE)
through Modulation of Crystallinity and Polymorphism. Polymer (Guildf).

[22] Su H., Strachan A., Goddard W. A. (2004). Density Functional Theory and Molecular
Dynamics Studies of the Energetics and Kinetics of Electroactive Polymers:
PVDF and P­(VDF-TrFE). Phys. Rev. B.

[23] Apelt S., Hohne S., Mehner E., Bohm C., Malanin M., Eichhorn K.-J., Jehnichen D., Uhlmann P., Bergmann U. (2022). Poly­(vinylidene
fluoride-*co*-trifluoroethylene) Thin Films after Dip-
and Spin-Coating. Macromol. Mater. Eng..

[24] Jia N., He Q., Sun J., Xia G., Song R. (2017). Crystallization Behavior
and Electroactive Properties of PVDF, P­(VDF-TrFE) and Their Blend
Films. Polym. Test.

[25] Guo D., Setter N. (2013). Impact of Confinement-Induced
Cooperative Molecular
Orientation Change on the Ferroelectric Size Effect in Ultrathin P­(VDF-TrFE)
Films. Macromolecules.

[26] Qian J., Jiang S., Wang Q., Yang C., Duan Y., Wang H., Guo J., Shi Y., Li Y. (2018). Temperature
Dependence of Piezo- and Ferroelectricity in Ultrathin P­(VDF-TrFE)
Films. RSC Adv..

[27] Du X., Zhao M., Chen G., Zhang X. (2015). Thickness Dependence
of Ferroelectric Properties for Ferroelectric Random Access Memory
Based on Poly­(Vinylidene Fluoride-Trifluoroethylene) Ultrathin Films. Ferroelectrics.

[28] Hong L., Soh A. K., Song Y. C., Lim L. C. (2008). Interface and Surface
Effects on Ferroelectric Nano-Thin Films. Acta
Mater..

[29] Dolynchuk O., Kahl R. T., Meichsner F., Much A. J., Pechevystyi A., Averkova A., Erhardt A., Thelakkat M., Thurn-Albrecht T. (2024). Controlling Crystal Orientation in
Films of Conjugated
Polymers by Tuning the Surface Energy. Macromolecules.

[30] Zhao C., Hong Y., Chu X., Dong Y., Hu Z., Sun X., Yan S. (2021). Enhanced Ferroelectric Properties of P­(VDF-TrFE) Thin
Film on Single-Layer Graphene Simply Adjusted by Crystallization Condition. Mater. Today Energy.

[31] Wu Y., Hsu S. L., Honeker C., Bravet D. J., Williams D. S. (2012). The Role
of Surface Charge of Nucleation Agents on the Crystallization Behavior
of Poly­(Vinylidene Fluoride). J. Phys. Chem.
B.

[32] Ritsema
van Eck G. C., Chiappisi L., de Beer S. (2022). Fundamentals and Applications
of Polymer Brushes in Air. ACS Appl. Polym.
Mater..

[33] Mansky P., Liu Y., Huang E., Russell T. P., Hawker C. (1997). Controlling Polymer-Surface
Interactions with Random Copolymer Brushes. Science (1979).

[34] Lundy R., Yadav P., Selkirk A., Mullen E., Ghoshal T., Cummins C., Morris M. A. (2019). Optimizing
Polymer Brush Coverage
To Develop Highly Coherent Sub-5 Nm Oxide Films by Ion Inclusion. Chem. Mater..

[35] Oria L., Ruiz de Luzuriaga A., Alduncin J. A., Perez-Murano F. (2013). Polystyrene
as a Brush Layer for Directed Self-Assembly of Block Co-Polymers. Microelectron. Eng..

[36] Lundy R., Yadav P., Prochukhan N., Giraud E. C., O’Mahony T. F., Selkirk A., Mullen E., Conway J., Turner M., Daniels S., Mani-Gonzalez P. G., Snelgrove M., Bogan J., McFeely C., O’Connor R., McGlynn E., Hughes G., Cummins C., Morris M. A. (2020). Precise
Definition of a “Monolayer Point” in Polymer Brush Films
for Fabricating Highly Coherent TiO2 Thin Films by Vapor-Phase Infiltration. Langmuir.

[37] Yadav P., Gatensby R., Prochukhan N., C. Padmanabhan S., Davó-Quiñonero A., Darragh P., Senthamaraikannan R., Murphy B., Snelgrove M., McFeely C., Singh S., Conway J., O’Connor R., McGlynn E., Lundy R., Morris M. A. (2022). Fabrication of High-κ
Dielectric Metal Oxide
Films on Topographically Patterned Substrates: Polymer Brush-Mediated
Depositions. ACS Appl. Mater. Interfaces.

[38] Prochukhan N., Davó-Quiñonero A., Alvarez-Fernandez A., Yadav P., Beloshapkin S., Keegan A. J., Lundy R., Morris M. A. (2025). Enhanced Binding of (3-Aminopropyl)­Triethoxysilane
to Polymer Brush-Coated Surfaces by Controlled Activation: Degradation,
Activation, and Functionalization. ACS Omega.

[39] Yu B., Chang B. S., Loo W. S., Dhuey S., O’Reilly P., Ashby P. D., Connolly M. D., Tikhomirov G., Zuckermann R. N., Ruiz R. (2024). Nanopatterned Monolayers of Bioinspired,
Sequence-Defined Polypeptoid Brushes for Semiconductor/Bio Interfaces. ACS Nano.

[40] Masuda T. (2024). Design of
Functional Soft Interfaces with Precise Control of the Polymer Architecture. Polym. J..

[41] Bocchiaro A. Lo, Avanzini E., Lorandi F., Benetti E. M. (2025). The Future
of Polymer
Brushes. Eur. Polym. J..

[42] Gómez-Rodríguez J. M., Horcas I., Gómez-Herrero J., Baro A. M., Colchero J., Fernández R. (2007). WSXM: A Software for Scanning Probe
Microscopy and a Tool for Nanotechnology. Rev.
Sci. Instrum..

[43] Hemmerle A., Aubert N., Moreno T., Kékicheff P., Heinrich B., Spagnoli S., Goldmann M., Ciatto G., Fontaine P. (2024). Opportunities and New Developments
for the Study of
Surfaces and Interfaces in Soft Condensed Matter at the SIRIUS Beamline
of Synchrotron SOLEIL. J. Synchrotron Radiat.

[44] María N., Le Goupil F., Cavallo D., Maiz J., Müller A. J. (2022). Effect
of the TrFE Content on the Crystallization and SSA Thermal Fractionation
of P­(VDF-Co-TrFE) Copolymers. Int. J. Mol. Sci..

[45] Rams J., López A. J., Sánchez M., Ureña A., Leal V., Sánchez-Mariscal B., Lafuente P. (2012). Application
of Atomic Force Microscopy to the Study of Blown Polyethylene Films. Polym. Test.

[46] Zhang J., Ebbens S., Chen X., Jin Z., Luk S., Madden C., Patel N., Roberts C. J. (2006). Determination
of
the Surface Free Energy of Crystalline and Amorphous Lactose by Atomic
Force Microscopy Adhesion Measurement. Pharm.
Res..

[47] Lanceros-Méndez S., Mano J. F., Costa A. M., Schmidt V. H. (2001). FTIR and DSC Studies
of Mechanically Deformed β-PVDF Films. Journal of Macromolecular Science, Part B.

[48] Lauchlan L., Rabolt J. F. (1986). Polarized Raman
Measurements of Structural Anisotropy
in Uniaxially Oriented Poly­(Vinylidene Fluoride) (Form I). Macromolecules.

[49] Kim K. J., Kim G. B. (1993). Curie Transition
and Piezoelectricity of the Blends
of a Ferroelectric VDF/TrFE Copolymer and PMMA. J. Appl. Polym. Sci..

[50] Mu X., Zhang H., Zhang C., Yang S., Xu J., Huang Y., Xu J., Zhang Y., Li Q., Wang X., Cao D., Li S. (2021). Poly­(vinylidene fluoride-trifluoroethylene)/Cobalt
Ferrite Composite Films with a Self-Biased Magnetoelectric Effect
for Flexible AC Magnetic Sensors. J. Mater.
Sci..

[51] Choi J., Lee K., Lee M., Kim T., Eom S., Sim J. H., Lee W. B., Kim Y. J., Park C., Kang Y. (2023). High β-Phase
Poly­(Vinylidene Fluoride) Using a Thermally Decomposable Molecular
Splint. Adv. Electron Mater..

[52] Park Y. J., Kang S. J., Lotz B., Brinkmann M., Thierry A., Kim K. J., Park C. (2008). Ordered Ferroelectric
PVDF-TrFE Thin Films by High Throughput Epitaxy for Nonvolatile Polymer
Memory. Macromolecules.

[53] Ye H., Kwon H. J., Ryu K. Y., Wu K., Park J., Babita G., Kim I., Yang C., Kong H., Kim S. H. (2024). Surface Engineering of High-k Polymeric
Dielectric
Layers with a Fluorinated Organic Crosslinker for Use in Flexible-Platform
Electronics. Nanoscale Adv..

[54] Mesforush S., Cazorla A., Melville H., Blanchard P., Klauk H., Zschieschang U., Zhang M., Fijahi L., Mas-Torrent M., Barrena E. (2025). Gate-Dielectric Surface Engineering
With Fluorinated Monolayers: Minimizing Contact Resistance and Nonidealities
in OFETs. Adv. Electron Mater..

[55] Kong G. D., Choi Y., Yoon H. J. (2025). Self-Assembled
Monolayers for Advanced
Energy Conversion Technologies. ACS Appl. Nano
Mater..

[56] Park Y. J., Kang S. J., Shin Y., Kim R. H., Bae I., Park C. (2011). Non-Volatile Memory Characteristics of Epitaxially Grown PVDF-TrFE
Thin Films and Their Printed Micropattern Application. Curr. Appl. Phys..

[57] Kim K. L., Lee W., Hwang S. K., Joo S. H., Cho S. M., Song G., Cho S. H., Jeong B., Hwang I., Ahn J. H., Yu Y. J., Shin T. J., Kwak S. K., Kang S. J., Park C. (2016). Epitaxial
Growth of Thin Ferroelectric Polymer Films on Graphene
Layer for Fully Transparent and Flexible Nonvolatile Memory. Nano Lett..

[58] Bai X., Li H., Chu X., Liu M., Li H., Wang H., Sun X., Yan S. (2024). The Tuning of Crystallization
Behavior of Ferroelectric
Poly­(Vinylidene Fluoride-co-trifluoroethylene). J. Polym. Sci..

[59] Lange H., Schmid F. (2002). Surface Anchoring on
Liquid Crystalline Polymer Brushes. Comput.
Phys. Commun..

[60] Khan M. A., Bhansali U. S., Alshareef H. N. (2011). Fabrication
and Characterization
of All-Polymer, Transparent Ferroelectric Capacitors on Flexible Substrates. Org. Electron.

[61] Wang C., Shen Q., Kou B. (2015). Crystallisation
Behaviours of Ferroelectric
P­(VDF-TrFE) Ultrathin Films on Different Substrates. Materials Research Innovations.

[62] Sommer J. U., Reiter G. (2005). The Formation of Ordered
Polymer Structures at Interfaces:
A Few Intriguing Aspects. Adv. Polym. Sci..

[63] Ruan L., Yao X., Chang Y., Zhou L., Qin G., Zhang X. (2018). Properties
and Applications of the β Phase Poly­(vinylidene fluoride). Polymers.

[64] Meng N., Ren X., Zhu X., Wu J., Yang B., Gao F., Zhang H., Liao Y., Bilotti E., Reece M. J., Yan H. (2020). Multiscale Understanding of Electric Polarization in Poly­(Vinylidene
Fluoride)-Based Ferroelectric Polymers. J. Mater.
Chem. C Mater..

[65] Shaheen A., Anwar N., Chen F., Chan Y., Xie H., Lee S.-L. (2025). Materials Interface Engineering: Impact of Interfacial
Molecular Orientation on Organic Electronic Devices. Adv. Funct Mater..

