# Supplementary material for: Engineering Surface Chemistry to Enhance Ferroelectric Phase Formation in Ultrathin PVDF-TrFE Films
Source: Macromolecules. 2026 Jan 14;59(2):974–85. doi: 10.1021/acs.macromol.5c03532 (PMC12854770; doi:10.1021/acs.macromol.5c03532)
Supplement: Supplementary file 1 [file ma5c03532_si_001.pdf]

-Supporting information-

Engineering Surface Chemistry to Enhance Ferroelectric Phase Formation in Ultrathin PVDF-TrFE Films

Andres Mosquera-Vallin,[1] Arnaud Hemmerle,[2] Jon Maiz,[1,3]* Alberto Alvarez-Fernandez.[1]*

[1] Centro de Fisica de Materiales (CFM-MPC), CSIC-EHU, 20018 Donostia - San Sebastian, Spain

[2] Synchrotron SOLEIL, L'Orme des Merisiers, Départementale 128, 91190 Saint-Aubin, France

[3] IKERBASQUE-Basque Foundation for Science, Plaza Euskadi 5, 48009 Bilbao, Spain

Email: jon.maizs@ehu.eus; alberto.alvarez@ehu.eus

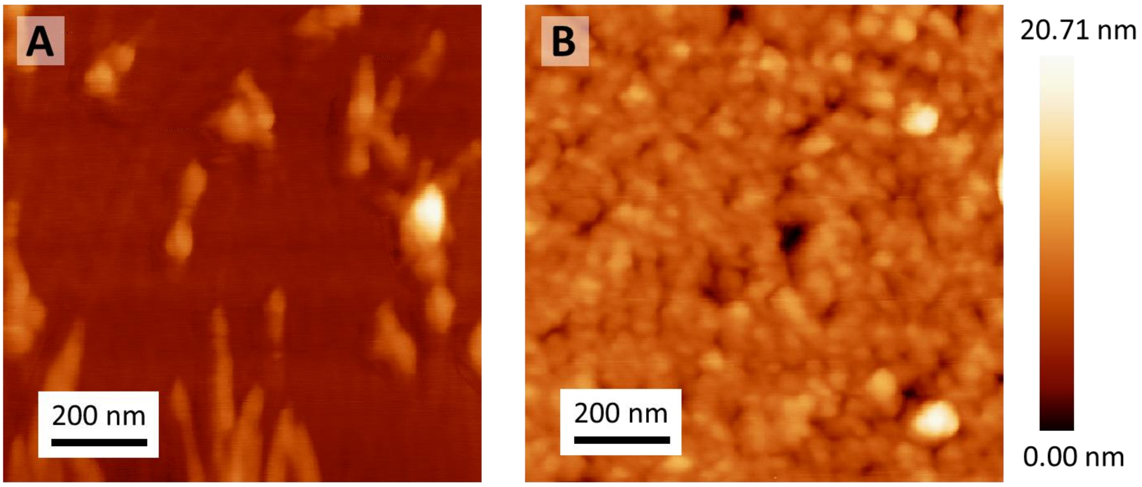

Figure S1. AFM micrographs of PVDF thin films deposited on (A) pristine Si and (B) Si–PS surfaces.

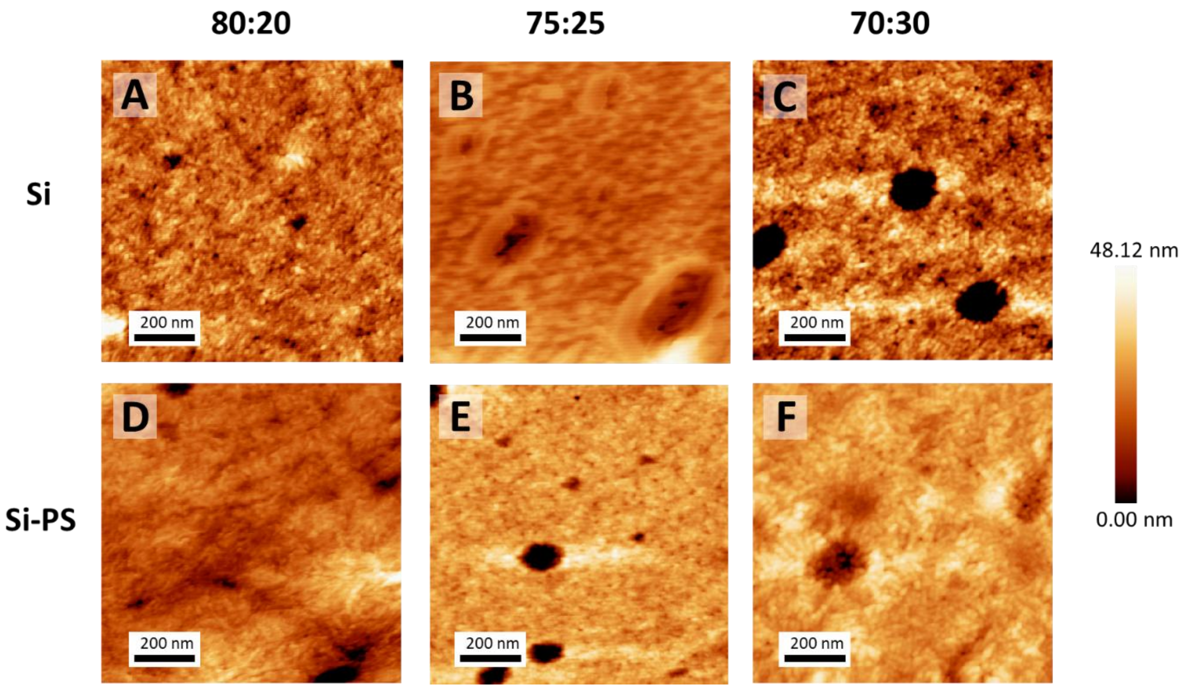

Figure S2. AFM topography images of the non-annealed PVDF-TrFE copolymer thin films with different VDF:TrFE ratios deposited on pristine silicon (A-C) and PS-grafted silicon (D-F). (A, D) 80:20; (B, E) 75:25; (C, F) 70:30.

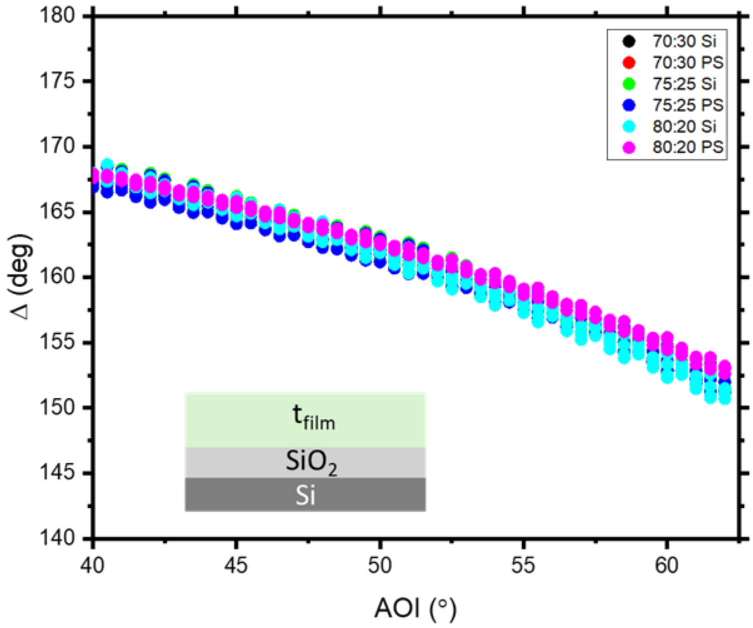

Figure S3. Ellipsometry measurements of PVDF-TrFE thin films with varying ratios (70:30, 75:25, and 80:20) deposited on Si and Si-PS substrates. The inset illustrates the schematic layer structure used for the fitting model (PVDF-TrFE thin film on SiO_2/Si).

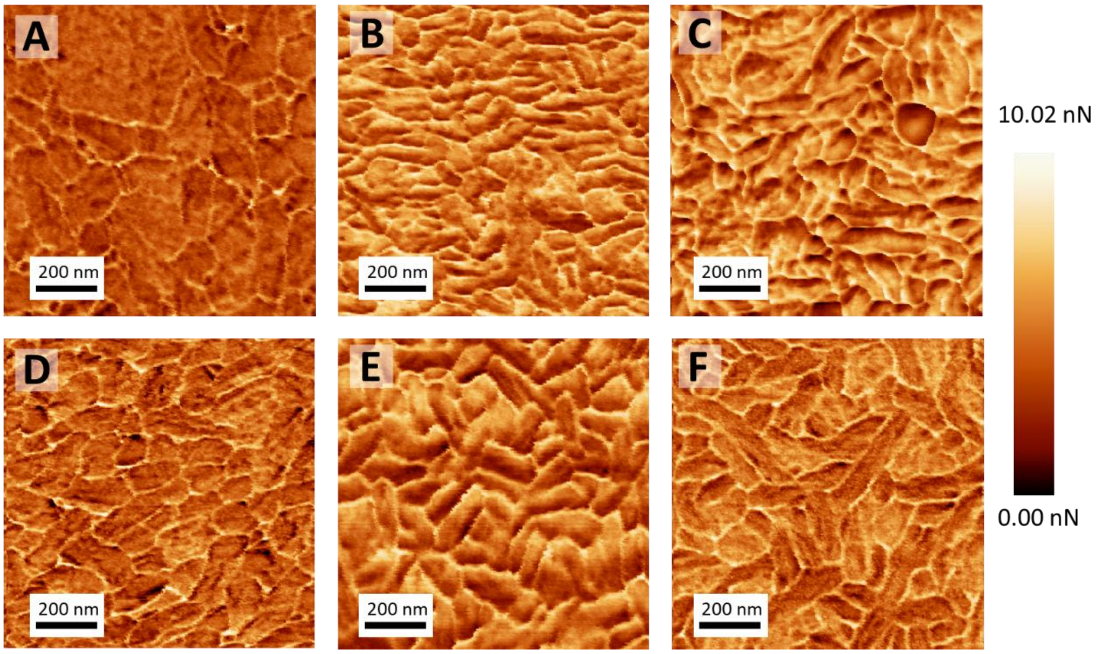

Figure S4. AFM adhesion force maps recorded concurrently with the topography in Fig. 3 for PVDF-TrFE 80:20 (A, D), 75:25 (B, E), and 70:30 (C, F) on pristine Si (top) and PS-grafted Si (bottom), respectively.

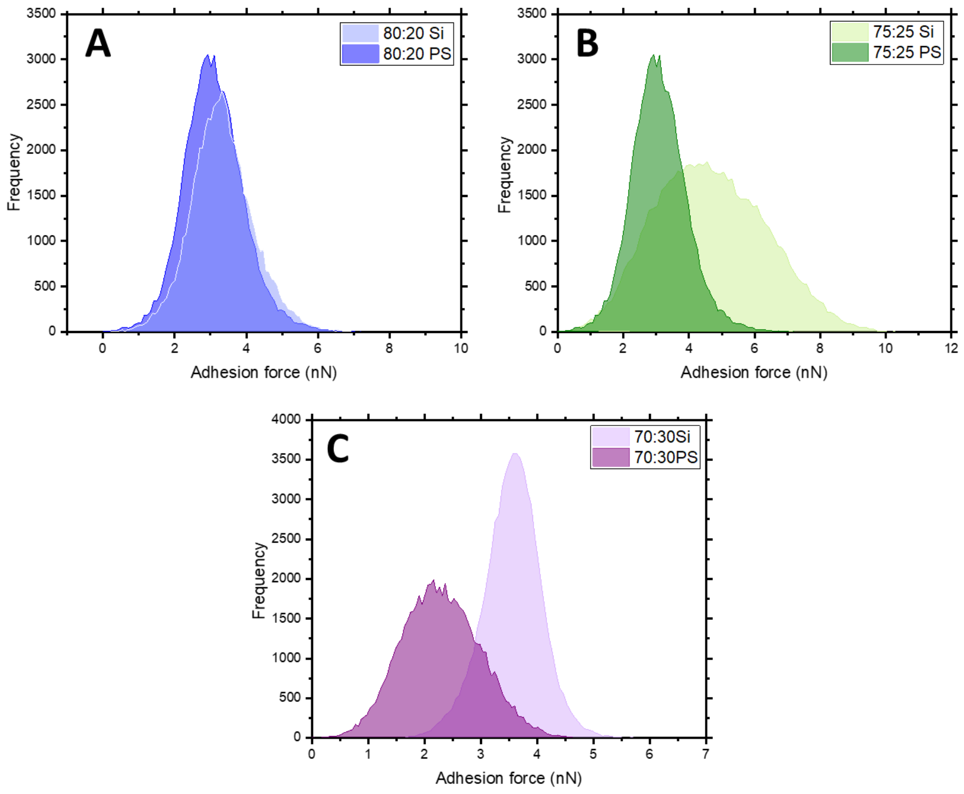

Figure S5. Adhesion force histograms of the images presented in Figure S4.

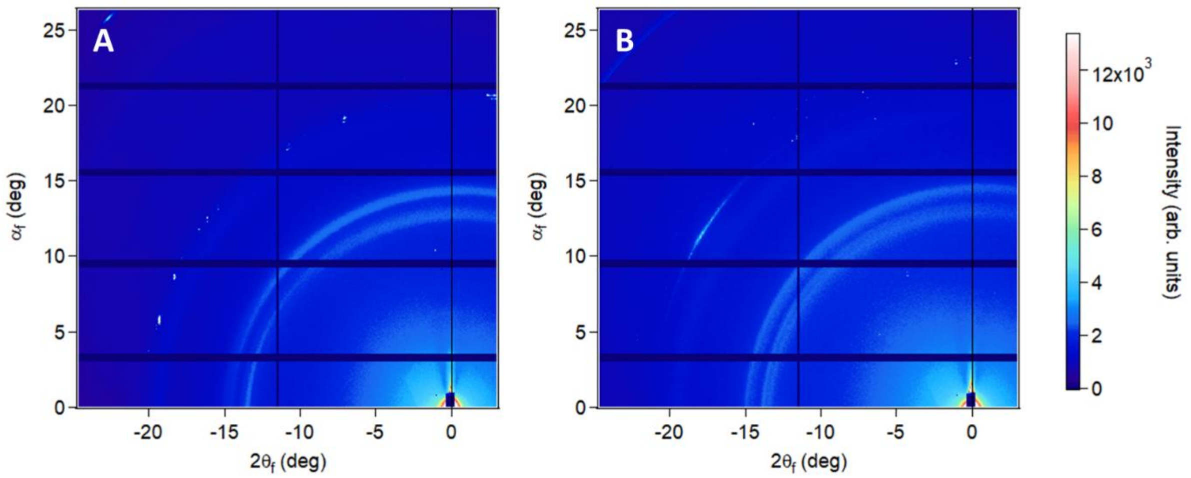

Figure S6. 2D GIWAXS patterns for PVDF films on A) Si and B) Si-PS surfaces.
